# Powder Spreading Dynamics and Process Optimization at a Heterogeneous Interface for Z-Direction Multi-Material Laser Powder Bed Fusion

**DOI:** 10.3390/ma19091762

**Published:** 2026-04-26

**Authors:** Zhaowei Xiang, Shuai Ma, Fulin Han, Ju Wang

**Affiliations:** School of Mechanical Engineering, Chongqing University of Technology, Chongqing 400054, China; hflhfllbj@163.com (F.H.); wjwangjuu@163.com (J.W.)

**Keywords:** multi-material additive manufacturing, laser powder bed fusion, heterogeneous interface, powder spreading process, discrete element method, spreading process optimization

## Abstract

This paper investigates the powder spreading process in a Z-direction multi-material fabrication system utilizing a blade. Focusing on 316L stainless steel and CuCrZr, a discrete element model was developed to simulate powder spreading at the heterogeneous material interface. The effects of spreading speed and theoretical layer thickness on the resulting powder bed quality were systematically examined. The results reveal that during spreading over a heterogeneous bed, the underlying powder exhibits an unsteady “forward-surging and rearward-suppressing” motion pattern, with inter-particle force chains displaying significant spatiotemporal fluctuations. Increasing the spreading speed exacerbates the disturbance and removal of the underlying powder, leading to a reduction in the deposited mass of CuCrZr and a deterioration in its distribution uniformity. Conversely, increasing the layer thickness effectively mitigates the mechanical disturbance of the underlying powder by the blade, significantly enhancing both the deposited mass of CuCrZr and its distribution uniformity. Further investigation demonstrates that employing a higher spreading speed in combination with a larger layer thickness can achieve a favorable powder bed quality while maintaining high spreading efficiency, thereby enabling a synergistic optimization of productivity and bed quality. This work elucidates the mesoscopic dynamic mechanisms governing the powder spreading process at Z-direction heterogeneous interfaces and provides a theoretical foundation for process optimization in multi-material laser powder bed fusion.

## 1. Introduction

Multi-material additive manufacturing (MM-AM) enables the precise spatial arrangement and integrated fabrication of multiple materials within a single component, allowing different regions to be tailored with specific materials to meet diverse functional requirements. This “on-demand material allocation” paradigm offers a novel technological pathway for the synergistic optimization of a component’s material composition, structural design, and resultant performance [1,2]. MM-AM not only holds the potential to surpass the performance limitations inherent in conventional homogeneous materials but also facilitates part consolidation, shortens manufacturing lead times, and enhances system reliability. Consequently, it represents a significant development frontier and a prominent research focus within the AM field [3,4].

MM-AM encompasses various process categories, including laser powder bed fusion (LPBF), directed energy deposition (DED), wire arc additive manufacturing (WAAM), and cold spraying [5,6,7]. Among these, LPBF, owing to its distinct advantages such as high forming precision, excellent surface finish, and capability to fabricate complex geometric features, demonstrates considerable promise for producing multi-metal components [8,9,10]. In this process, a blade or roller spreads a thin layer of powder onto a substrate, after which a high-energy laser beam selectively melts the powder according to a predefined path, building the three-dimensional object layer by layer. To achieve multi-material capability, researchers have developed various modifications to conventional single-material spreading systems, which can be broadly categorized into blade-based, ultrasound-assisted, electrophotography-based, and multi-technique hybrid systems [11,12,13]. Blade-based systems typically achieve heterogeneous material distribution along the build direction (Z-direction) by sequentially spreading from distinct powder reservoirs. Ultrasound-assisted delivery utilizes high-frequency vibrations to precisely deposit powder from fine nozzles, enabling patterned powder placement within a single layer and offering the possibility of constructing complex heterogeneous interfaces. Electrophotography-based methods, inspired by laser printing principles, achieve high-resolution selective powder transfer via electrostatic forces, rendering them particularly suitable for fabricating fine functionally graded structures. Although blade-based spreading is limited to layer-by-layer heterogeneity along the Z-direction, its simplicity, high reliability, and suitability for unidirectional multi-material components have made it a research hotspot and led to some industrial adoption [14].

Powder spreading is a critical step in the LPBF process, as the quality of the spread layer directly influences subsequent melting stability and, ultimately, the performance of the final part [15]. Direct experimental observation of powder dynamics during spreading is challenging due to the typical particle size of tens of micrometers. The Discrete Element Method (DEM) provides a powerful tool to simulate the motion, collision, and packing behavior of individual particles at the microscale, offering valuable insights into powder bed formation mechanisms and the influence of spreading parameters on bed quality [16,17]. In recent years, DEM has been extensively employed to study powder spreading. Pioneering work by Parteli et al. [18], Chen et al. [19,20,21], and Nan et al. [22,23] established foundational DEM models for LPBF spreading processes, revealing particle flow behavior through combined numerical simulation and experimental validation. Building upon this, researchers have extended DEM applications to multi-material systems. For mixed powder systems, studies by Chen et al. [24], Yao et al. [25], Zhang et al. [26], and Tan et al. [27] developed DEM models for spreading multi-material mixtures, elucidating the influence of particle size, volume fraction, and process parameters on packing density and uniformity. Wang et al. [28] investigated scenarios where material composition varies perpendicular to the spreading direction, studying particle segregation during the spreading of two metal powders with significantly different densities. However, existing research predominantly focuses on single materials or homogeneous mixtures, leaving the spreading behavior at the heterogeneous interface region in Z-direction multi-material fabrication relatively unexplored.

This paper addresses this gap by focusing on a Z-direction multi-material fabrication process utilizing a doctor blade. A DEM simulation model is developed to investigate powder spreading dynamics on a pre-existing heterogeneous powder bed. The influence of key process parameters, specifically spreading speed and theoretical layer thickness, on the quality of the resulting powder bed is systematically analyzed, aiming to provide a scientific basis for process optimization in multi-material LPBF.

## 2. Modeling of the Spreading Process

### 2.1. Simulation Setup for Z-Direction Multi-Material Spreading

**Figure 1:**
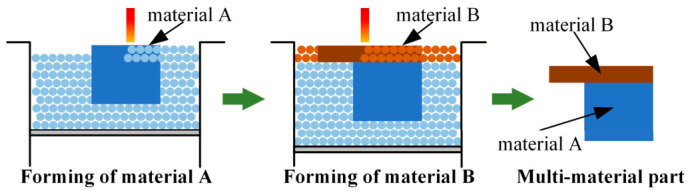
Schematic of the multi-material component fabrication process.

In the Z-direction multi-material fabrication process based on doctor blade spreading, material A (potentially constituting several layers) is first fabricated. Subsequently, material B is spread and melted via laser (also potentially for multiple layers), ultimately yielding the multi-material component, as illustrated schematically in Figure 1.

**Figure 2:**
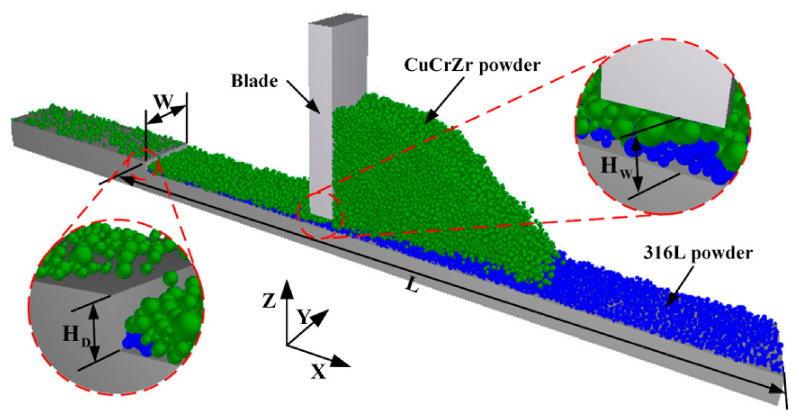
DEM simulation model of the multi-material spreading process.

During the transition from fabricating one material to spreading the next, powder particles of the two materials inevitably undergo complex interactions due to their mesoscopic scale and the dynamic nature of spreading. This can lead to interpenetration and mixing, which significantly impacts subsequent processing and the performance of the bonding region in the final component. To investigate these interactions, a DEM simulation model was established, as shown in Figure 2. The effective spreading length L is 7 mm, and the width W is 0.5 mm. Periodic boundary conditions are applied in the Y-direction to eliminate sidewall effects and enhance computational efficiency. The theoretical spreading layer thickness for the underlying material $H_{D}$ is set to 200 µm, but due to the dynamic relaxation of the powder bed after blade passage [29,30], the actual measured thickness of the underlying powder bed is approximately 150 µm. The nominal theoretical spreading layer thickness for the upper material is $H_{U}$, where $H_{U}=H_{W}-H_{D}$. The default value of $H_{U}$ is 50 µm, but the actual spreading layer thickness will be substantially larger because the initial underlying bed is not fully dense [29]. Here, $H_{W}$ is the distance between the bottom of the multi-material powder bed and the blade when spreading the upper powder material. The study employs the common multi-material combination of 316L stainless steel (316L SS) and CuCrZr, with 316L SS as the underlying powder and CuCrZr as the upper spreading powder.

**Figure 3:**
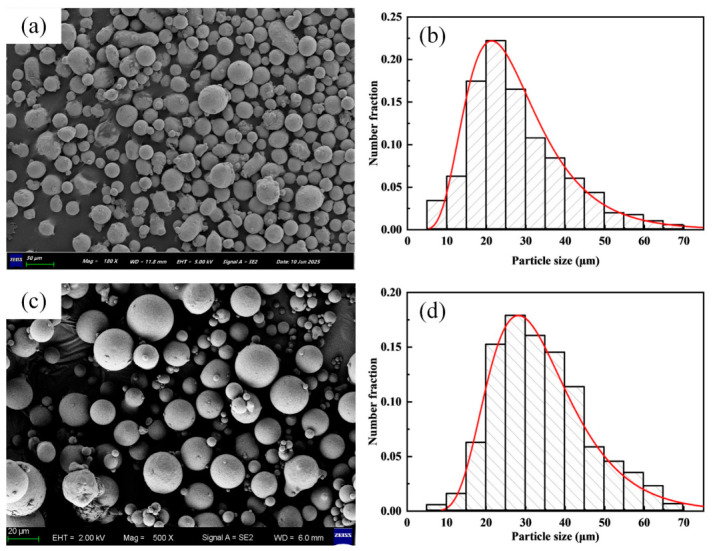
Powder characteristics. (**a**) SEM image of 316L SS powder. (**b**) Particle size distribution of 316L SS powder. (**c**) SEM image of CuCrZr powder. (**d**) Particle size distribution of CuCrZr powder.

The powder particles are representative of actual additive manufacturing feedstocks. The size distribution for both materials falls predominantly within 15–53 µm. The 316L SS powder was sourced from Avimetal AM (Xuzhou) Tech Co. (Xuzhou, China), and the CuCrZr powder from Jiangsu Vilory Advanced Materials Co., Ltd (Xuzhou, China). Scanning electron microscope (SEM) images and the corresponding particle size distributions are shown in Figure 3.

### 2.2. Discrete Element Method Formulation

The Discrete Element Method is employed to simulate the powder spreading process over the heterogeneous powder bed. The translational and rotational motions of each particle are governed by Newton’s second law:

(1) $$m_{i}\frac{dv_{i}}{dt}=\sum_{j}\left(F_{ij}^{n}+F_{ij}^{t}\right)+m_{i}g$$

(2) $$I_{i}\frac{d\omega_{i}}{dt}=\sum_{j}\left(T^{t}+T^{r}\right)$$

where $m_{i}$, $I_{i}$, $v_{i}$ and $\omega_{i}$ represent the mass, moment of inertia, translational velocity, and angular velocity of particle i, respectively; g is the gravitational acceleration vector; $F_{ij}^{n}$ and $F_{ij}^{t}$ are the normal and tangential contact forces exerted on particle i by particle j; $T^{t}$ and $T^{r}$ are the torques arising from tangential forces and rolling resistance, respectively [31].

The normal and tangential contact forces are calculated based on the Hertz–Mindlin contact model, incorporating elastic and damping components:

(3) $$F_{ij}^{n}=\frac{4}{3}E^{*}\sqrt{R^{*}}\delta_{n}^{3/2}-2\sqrt{\frac{5}{6}}\beta \sqrt{S_{n}m^{*}}v_{ij}^{n}$$

(4) $$F_{ij}^{t}=-\min\left[S_{t}\delta_{t}+2\sqrt{\frac{5}{6}}\beta \sqrt{S_{t}m^{*}}v_{ij}^{t},\mu_{s}F_{ij}^{n}\right]$$

(5) $$T^{t}=R_{i}\times F_{ij}^{t}$$

(6) $$T^{r}=-\mu_{r}R_{i}F_{ij}^{n}{\bar{\omega }}_{i}$$

In these equations, E and R are the Young’s modulus and radius of the contacting particles; $\delta_{n}$ and $\delta_{t}$ are the normal and tangential overlaps; $v_{ij}^{n}$ and $v_{ij}^{t}$ are the normal and tangential components of the relative velocity; $\mu_{s}$ and $\mu_{r}$ are the sliding and rolling friction coefficients; and ${\bar{\omega }}_{i}$ is the unit angular velocity vector. The intermediate variables representing equivalent properties (β, $S_{n}$, $S_{t}$) are defined as follows [32]:

(7) $$\beta =-\frac{\ln\left(e\right)}{\sqrt{\ln^{2}\left(e\right)+\pi^{2}}}$$

(8) $$S_{n}=2E^{*}\sqrt{R^{*}\delta_{n}}$$

(9) $$S_{t}=8G^{*}\sqrt{R^{*}\delta_{n}}$$

where e is the restitution coefficient, $E^{*}$ is the equivalent Young’s modulus, $G^{*}$ is the equivalent shear modulus, $R^{*}$ is the equivalent radius, and $m^{*}$ is the equivalent mass, defined as [33]:

(10) $$E^{*}={\left(\frac{1-\sigma_{i}^{2}}{E_{i}}+\frac{1-\sigma_{j}^{2}}{E_{j}}\right)}^{-1}$$

(11) $$G^{*}={\left(\frac{2-\sigma_{i}}{G_{i}}+\frac{2-\sigma_{j}}{G_{j}}\right)}^{-1}$$

(12) $$R^{*}=\frac{R_{i}R_{j}}{R_{i}+R_{j}}$$

(13) $$m^{*}=\frac{m_{i}m_{j}}{m_{i}+m_{j}}$$

where σ is Poisson’s ratio. Given the small particle size characteristic of LPBF feedstocks, inter-particle adhesion forces significantly influence dynamic behavior during spreading. Therefore, the Hertz–Mindlin model is augmented with the Johnson–Kendall–Roberts (JKR) cohesion theory to account for these adhesive effects [34]. The normal force is then expressed as:

(14) $$F_{JKR}=\frac{4E^{*}}{3R^{*}}\alpha^{3}-{\left(8\pi \Gamma E^{*}\alpha^{3}\right)}^{1/2}$$

where Γ is the effective surface energy [35]:

(15) $$\Gamma =\gamma_{i}+\gamma_{j}-\gamma_{ij}$$

where $\gamma_{i}$, $\gamma_{ij}$ are the surface energies of particles i and j, respectively, and $\gamma_{ij}$ is their interfacial surface energy. For particles of the same material, $\gamma_{ij}=0$. The contact radius α is related to the normal overlap $\delta_{n}$ by the following implicit equation [36]:

(16) $$\delta_{n}=\frac{\alpha^{2}}{R^{*}}-\sqrt{\frac{4\pi \Gamma \alpha }{E^{*}}}$$

### 2.3. Simulation Parameters and Model Validation

The physical properties used for 316L SS and CuCrZr in the DEM simulations are summarized in Table 1 [19,37]. The selection of an appropriate time step is crucial for the stability and accuracy of DEM simulations. The Rayleigh critical time step provides a key reference [38]:

(17) $$\Delta t=\pi {\left[\frac{R}{0.163\sigma +0.877}\sqrt{\frac{\rho }{G}}\right]}_{\min}$$

where G is the material’s shear modulus and ρ is the density. As indicated by Equation (17), the critical time step decreases with increasing shear modulus. To permit a larger time step and reduce computational cost, the shear modulus used in the simulations was scaled down to 1/100 of its actual value, a common practice in DEM simulations of cohesive powders [39]. Accordingly, a fixed time step of $2.5\times 10^{-8}$ s was employed. It should be noted that the surface energy values listed in Table 1 are not the intrinsic thermodynamic surface energies of the metals, but rather effective, calibrated parameters determined jointly by the reduction in the shear modulus (to 1/100 of the physical value) and the calibration of other key parameters (e.g., rolling friction coefficient, restitution coefficient).

**Figure 4:**
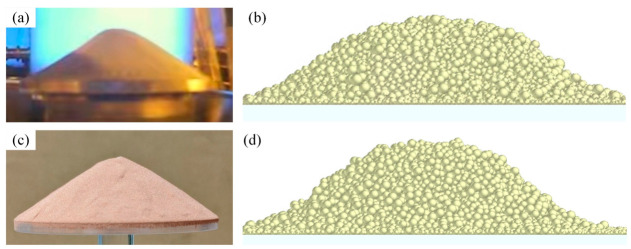
Angle of repose. (**a**) Experimental measurement for 316L SS. (**b**) Simulated measurement for 316L SS. (**c**) Experimental measurement for CuCrZr. (**d**) Simulated measurement for CuCrZr.

To validate the chosen parameters and the overall DEM model, angle of repose simulations was performed for each powder type and compared with experimental measurements, as shown in Figure 4. The experimentally measured angle of repose for 316L stainless steel is about 30.0°, which is close to the data obtained from experiments by other researchers [40,41], and in good agreement with the simulation results. For CuCrZr, the experimental angle was about 35°, which agreed well with the simulated value. This good agreement confirms the reliability of the calibrated model parameters.

## 3. Results and Discussion

### 3.1. Dynamics of the Heterogeneous Powder Spreading Process

**Figure 5:**
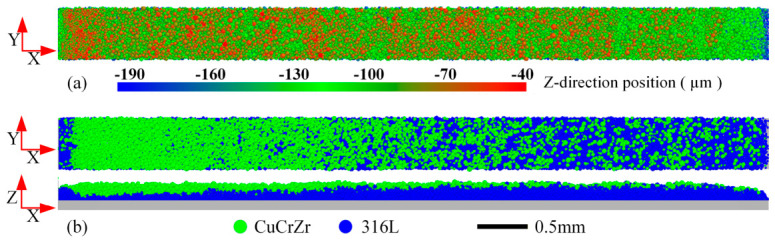
(**a**) Particle distribution of the underlying powder bed before spreading the upper CuCrZr powder, and (**b**) final particle distribution on the powder bed after spreading (v = 0.1 m/s, H = 50 µm).

Figure 5 shows the particle distribution of the underlying powder bed before spreading the upper CuCrZr powder, and the final particle distribution on the powder bed after spreading with a blade speed v = 0.1 m/s and a theoretical layer thickness H = 50 µm. Significant spatial non-uniformity is evident along the spreading direction (X-direction). Near the left end (the starting region), CuCrZr particles are densely deposited, achieving a local layer thickness of approximately 100 µm. In this region, the number of 316L SS particles is markedly reduced, with their coverage far below the initial pre-spread state (approx. 150 µm). Moving towards the right end, the amount of deposited CuCrZr gradually decreases, the particles become more dispersed, and the distribution eventually appears to stabilize.

This anomalous deposition pattern arises from the initial interaction between the spreading powder and the stationary underlying bed. At the start of spreading CuCrZr, the pre-spread 316L SS particles are static and exert resistance on the advancing front, causing CuCrZr accumulation. Simultaneously, the advancing CuCrZr pushes these 316L SS particles rightward. However, without replenishment from upstream, a particle-sparse zone forms in the underlying layer just ahead of the accumulation. As spreading continues towards the right, a dynamic equilibrium is eventually established: 316L SS particles displaced from downstream zones are continuously replenished by particles set in motion from upstream, and no further large-scale anomalies develop. Given this study’s focus on the fundamental spreading dynamics at a dissimilar material interface, subsequent analyses will concentrate on the right-hand region where quasi-steady conditions prevail, avoiding the initial transients.

**Figure 6:**
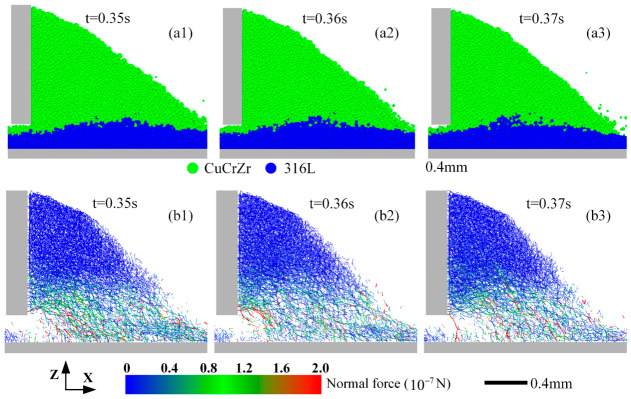
Snapshots of the spreading process at different times. Particle distribution showing material positions at (**a1**) t = 0.35 s, (**a2**) t = 0.36s, (**a3**) t= 0.37s; Corresponding inter-particle contact force chains at (**b1**) t = 0.35 s, (**b2**) t = 0.36s, (**b3**) t= 0.37s.

Figure 6 illustrates the relative positions and inter-particle contact force chains at different times during spreading. Figure 6(a1–a3) shows that the underlying 316L SS and the spreading CuCrZr generally remain stratified without deep interpenetration, although the interface is not perfectly flat. The advancing CuCrZr causes a slight upward bulge in the underlying 316L SS layer, and some particles at this perturbed interface exhibit limited mixing. Figure 6(b1–b3) reveals the contact force network. Significant forces develop within and near the interaction zone between the two materials, though the force chains appear relatively loose and discontinuous. In the upper region of the spreading powder pile (primarily CuCrZr), inter-particle forces are lower, but the force chains are denser, indicating closer packing. Near the blade’s bottom edge, force chains are predominantly oriented in the forward-downward direction (+X, −Z, i.e., ↘). At the very front of the pile, some strong force chains with a forward-upward orientation (+X, +Z, i.e., ↗) are observed. Crucially, the intensity and spatial distribution of force chains fluctuate significantly over time, highlighting the stochastic nature of particle interactions during spreading. This temporal randomness correlates well with the spatial randomness observed in the final particle distribution (Figure 5).

Under the continuous pushing action of the blade, the inter-particle contact network constantly reorganizes—the formation of strong force chains corresponds to a local “locked” state of particles, while the collapse of force chains releases particles, allowing them to re-engage in flow. This intermittent process of force chain formation and collapse is a fundamental characteristic distinguishing granular systems from continuous media, and serves as the microscopic origin of the randomness observed in the final powder bed distribution [39]. The spatiotemporal fluctuations of force chains reflect the self-organization behavior of the granular system under dynamic perturbation.

**Figure 7:**
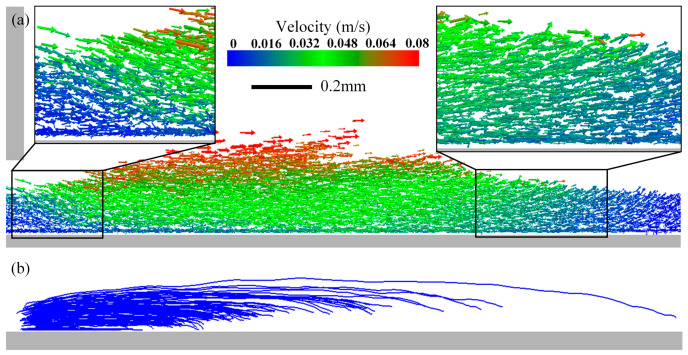
Motion of underlying 316L SS powder during CuCrZr spreading. (**a**) Velocity field. (**b**) Representative trajectories of individual 316L SS particles.

Figure 7 depicts the motion of underlying 316L SS particles induced by the spreading of upper CuCrZr particles. Figure 7a shows the velocity field. Although initially stationary, the 316L SS particles are set in motion through direct contact with the advancing CuCrZr and indirect shearing from the blade. Near the blade, 316L SS particles are pushed forward and downward. At the leading edge of the spreading pile, they are pushed forward and upward. This behavior is consistent with the particle trajectories exemplified in Figure 7b. The underlying particles exhibit a characteristic “forward-surging and rearward-suppressing” motion: particles at the front are lifted and carried forward, while those closer to the blade are pushed forward and downward into the bed. This observed motion aligns well with the orientation of force chains seen near the blade and at the pile front in Figure 6(b1–b3).

This phenomenon can be further understood from the particle velocity field. In front of the blade, particle velocity is not uniform: particles near the blade, directly pushed, move faster forward, while those farther away are driven downward by gravity. When the upper powder flows, the velocity gradient at the interface with the underlying powder induces motion in the lower particles: at the front edge of the pile, the velocity gradient direction causes underlying particles to be “lifted” and entrained into the flow (forward-surging); at the rear edge near the blade, the velocity gradient direction causes underlying particles to be “pressed” into the bed (rearward-suppressing). This non-uniform velocity field is the direct cause of the complex motion observed in the underlying particles [42].

**Figure 8:**
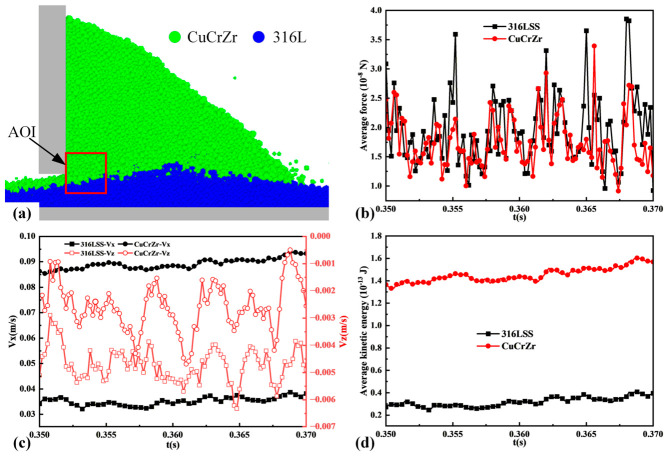
Time-resolved analysis of particle behavior within the moving AOI. (**a**) Definition and location of the AOI. (**b**) Average total force on particles. (**c**) Average X- and Z-direction velocities. (**d**) Average kinetic energy.

To quantitatively analyze particle dynamics, a moving area of interest (AOI) was defined directly in front of the blade, as shown in Figure 8a. The AOI is a rectangular region with dimensions of 400 μm in both the X- and Z-directions, extending through the entire model in the Y-direction. Its X-direction starts at the front tip of the blade, and it is symmetrically distributed about the bottom of the blade in the Z-direction. Figure 8b presents the average total force on particles within the AOI over time. Both particle types experience significant force fluctuations, and their fluctuations exhibit a notable synchronicity, indicating strong, time-varying coupling. Figure 8c shows the X- and Z-direction velocity components averaged over the AOI. The X-velocity dominates, confirming the blade’s primary role in driving forward motion. Velocities fluctuate, and again, the fluctuations for 316L SS and CuCrZr appear correlated. Figure 8d plots the average kinetic energy within the AOI, which correlates with the velocity fluctuations. The dynamic data from the AOI confirms that particle interactions during spreading are intense, fluctuating, and coupled between the two material types. This leads to the inherently variable deposition behavior and final bed heterogeneity observed in Figure 5 and Figure 6.

The synchronized fluctuations of particle forces and velocities indicate that the 316L SS and CuCrZr particles within the AOI are in a state of strong dynamic coupling—the flow of the upper CuCrZr directly drives the motion of the underlying 316L SS, while the presence of the underlying 316L SS, in turn, reacts back on the forces and motion of the upper particles. This inter-layer coupling is a core feature distinguishing heterogeneous interface spreading from single-material spreading, and is the fundamental reason for the complex dynamic behavior observed in the interfacial region [43].

### 3.2. Influence of Spreading Speed

**Figure 9:**
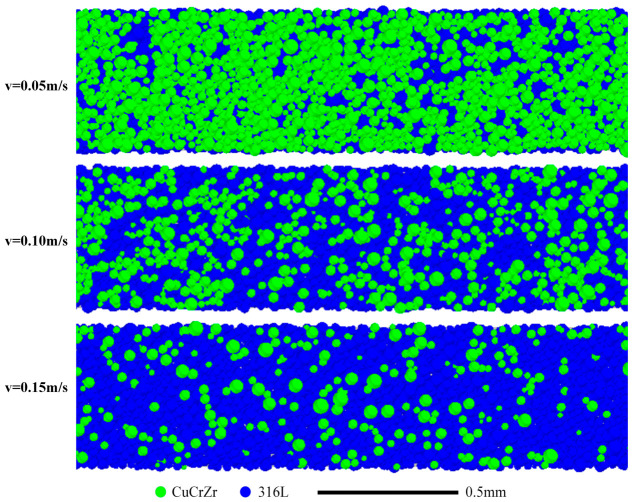
Powder bed particle distribution after spreading at different speeds.

Figure 9 shows the final powder bed morphology after spreading at different speeds (0.05, 0.10, and 0.15 m/s). Spreading speed profoundly affects bed quality. At the lowest speed (0.05 m/s), a relatively large amount of CuCrZr is deposited, but its distribution is highly uneven, featuring noticeable bare patches devoid of CuCrZr. Increasing the speed to 0.10 m/s reduces the overall deposited CuCrZr, and the distribution remains non-uniform. At the highest speed (0.15 m/s), only a sparse scattering of CuCrZr particles is deposited on the bed.

**Figure 10:**
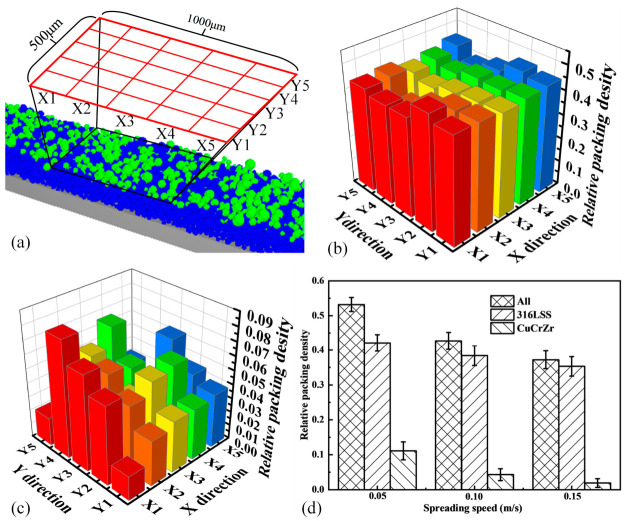
Quantitative analysis of powder bed density. (**a**) Schematic of the 5 × 5 sampling grid used for local relative packing density calculation. (**b**) Spatial map of relative density for all particles (v = 0.1 m/s). (**c**) Spatial map of relative density for CuCrZr particles only (v = 0.1 m/s). (**d**) Effect of spreading speed on the average relative density of all particles, and 316L SS-only and CuCrZr-only.

The packing density and its spatial uniformity are key metrics for evaluating powder bed quality. To quantify these, a relative packing density, φ, was defined based on a local evaluation volume:

(18) $$\varphi =\frac{\sum V_{P}}{L_{X}L_{Y}H_{Z}}$$

where $\Sigma V_{P}$ is the total particle volume within a sub-volume of in-plane dimensions $L_{X}$ and $L_{Y}$, and height $H_{Z}$. Based on particle Z-coordinate statistics (mainly −200 to 0 µm), $H_{Z}$ was set to 200 µm. To assess spatial uniformity, the central region of the bed (1000 µm × 500 µm) was divided into a 5 × 5 grid of 25 sub-volumes, as illustrated in Figure 10a. φ was calculated for each sub-volume. Figure 10b,c show the spatial distribution of φ for all particles and for CuCrZr particles only, respectively, at v = 0.1 m/s. The significant variation in φ across the grid confirms the poor uniformity visually observed in Figure 9. Figure 10d shows the average relative packing density for all particles, and for 316L SS-only and CuCrZr-only at different spreading speeds. Increasing speed has a detrimental effect on both materials. The CuCrZr density decreases substantially (from ~0.11 to ~0.02), indicating that fewer CuCrZr particles are retained on the bed. Simultaneously, the density of the pre-spread 316L SS also decreases (from ~0.42 to ~0.35), showing that higher spreading speeds increasingly disturb and remove the underlying powder.

**Figure 11:**
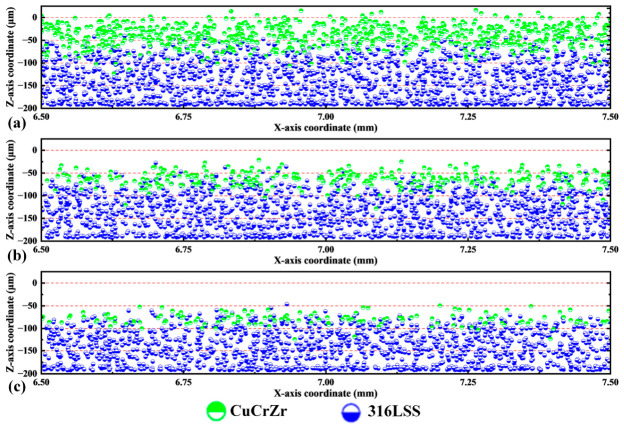
XZ plane projection of all particles in the final powder bed at different spreading speeds. (**a**) v = 0.05 m/s. (**b**) v = 0.10 m/s. (**c**) v = 0.15 m/s.

The vertical (Z-direction) distribution of particles provides further insight. Figure 11 projects particle positions onto the XZ plane for the three spreading speeds. At 0.05 m/s, a distinct layer of CuCrZr particles is visible primarily between Z ≈ −50 and 0 µm, with relatively few 316L SS particles in this upper zone. At 0.10 m/s and 0.15 m/s, the deposited CuCrZr layer is thinner and located deeper (mainly between −100 and −50 µm), and this zone is heavily intermixed with 316L SS particles. In practical production, the varying demands for material composition and properties across different sections of the target part, coupled with the influence of particle composition on forming quality, dictate the need for a relatively stable material composition in the intended forming area or component. Based on the foregoing analysis, this intermixing, undesirable for maintaining sharp material interfaces, is exacerbated at higher speeds. The lower speed (0.05 m/s) yields a higher-quality bed with a purer surface layer of the desired material (CuCrZr). For an actual Z-direction multi-material formed part, the fusion condition of its heterogeneous material interfaces is comprehensively affected by the quality of the powder bed heterogeneous interface, the number of heterogeneous interface layers, and the forming parameters [44,45,46]. To achieve good heterogeneous interface forming quality, comprehensive regulation of the powder bed heterogeneous interface characteristics and forming parameters is required.

**Figure 12:**
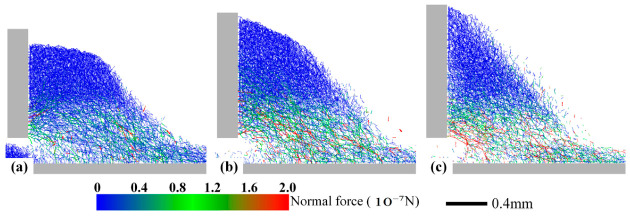
Inter-particle contact force chains during spreading at different speeds. (**a**) v = 0.05 m/s. (**b**) v = 0.10 m/s. (**c**) v = 0.15 m/s.

The underlying mechanism can be inferred from the contact force networks during spreading, shown in Figure 12. Higher spreading speeds intensify particle interactions, leading to the formation of stronger force chains, particularly across the material interface. This strong mechanical interaction promotes the entrainment and removal of underlying 316L SS particles and hinders the stable deposition of incoming CuCrZr. The sparser force chains observed at higher speeds also indicate a more dispersed, less stable particle configuration in front of the blade, which correlates with the reduced particle deposition. This provides a valid explanation for the distribution of particle coordinate projections in the XZ-plane of the powder bed under different spreading speeds, as shown in Figure 11.

The influence of speed can be further understood from the perspective of the force chain network. At low speed (0.05 m/s), the force chain network is relatively dense and stable, with sustained effective contacts between particles, facilitating the layer-by-layer deposition of upper particles and the stable anchoring of lower particles. As speed increases, the energy input from the blade rises, causing the force chain network to become sparser and more dynamic—strong force chains form frequently but also collapse rapidly, shortening the effective contact time between particles. This unstable contact state makes it difficult for upper particles to “anchor” on the bed surface, while simultaneously increasing the probability of underlying particles being entrained and removed, leading to reduced deposition and increased inter-layer mixing [47].

### 3.3. Influence of Layer Thickness

**Figure 13:**
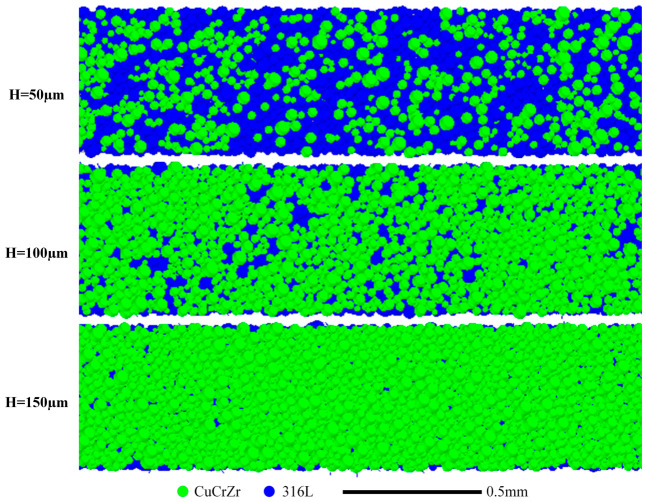
Powder bed particle distribution after spreading with different theoretical layer thicknesses (v = 0.1 m/s).

The theoretical layer thickness H is another critical parameter. Figure 13 shows the final powder bed for H = 50, 100, and 150 µm at a fixed spreading speed of 0.1 m/s. Increasing H from 50 to 100 µm results in significantly more CuCrZr deposition, but the distribution remains poor, with prominent voids. A further increase to 150 µm yields a substantial improvement: CuCrZr deposition is abundant and notably more uniform, with no large voids visible.

**Figure 14:**
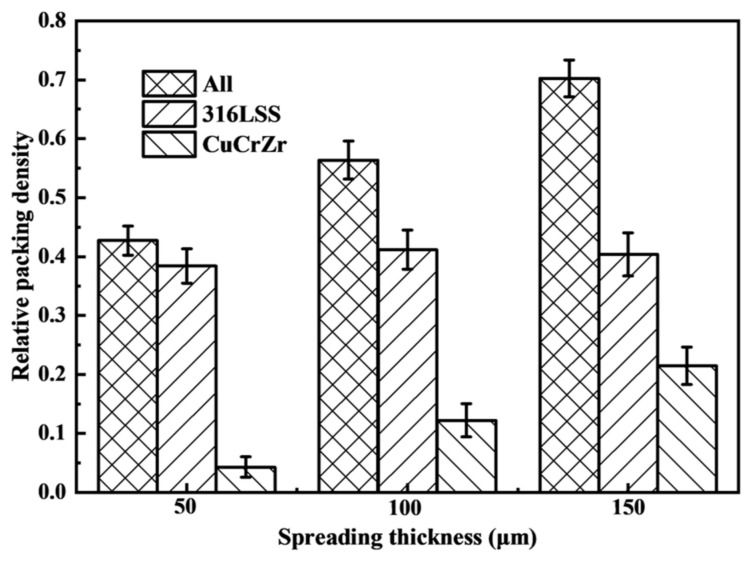
Effect of theoretical layer thickness on the average relative packing density of all particles, and 316L SS-only and CuCrZr-only particles (v = 0.1 m/s).

The quantitative effect on packing density is shown in Figure 14. The relative packing density of the powder bed (all particles) increases markedly with H, from ~0.43 at 50 µm to ~0.70 at 150 µm. This increase is almost entirely attributable to the increased deposition of CuCrZr, whose relative density rises from ~0.04 to ~0.21. The density of the underlying 316L SS remains relatively constant (~0.38–0.41), suggesting that a larger layer thickness primarily provides more space for the incoming powder to deposit without significantly disturbing the pre-existing layer.

**Figure 15:**
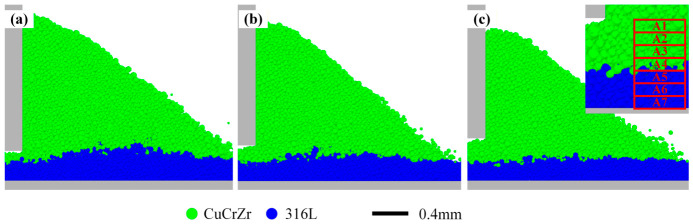
Snapshots during spreading showing particle distribution for different theoretical layer thicknesses. (**a**) H = 50 µm. (**b**) H = 100 µm. (**c**) H = 150 µm.

Snapshots during spreading (Figure 15) illustrate the mechanism. With a smaller gap (e.g., 50 µm), the blade and the advancing CuCrZr powder directly impinge on and displace the underlying 316L SS particles. As H increases, a thicker buffer zone of CuCrZr particles forms between the blade and the underlying 316L SS layer. This buffer absorbs much of the mechanical disturbance, shielding the underlying layer and providing a quiescent zone for incoming particles to settle.

This “shielding effect” can be understood from the perspective of momentum transfer between particles. When the layer thickness is small (50 µm), the upper CuCrZr layer is thin, allowing the driving force applied by the blade to be effectively transmitted to the interfacial region, directly driving the motion of underlying 316L SS particles. As the layer thickness increases, the upper powder layer thickens, lengthening the transmission path of the driving force, which dissipates through collisions and friction among multiple particle layers [48]. When the layer thickness reaches 150 µm, the driving force transmitted to the interface is substantially attenuated, becoming insufficient to significantly disturb the underlying particles, thus forming an effective “shield” for the lower powder layer.

**Figure 16:**
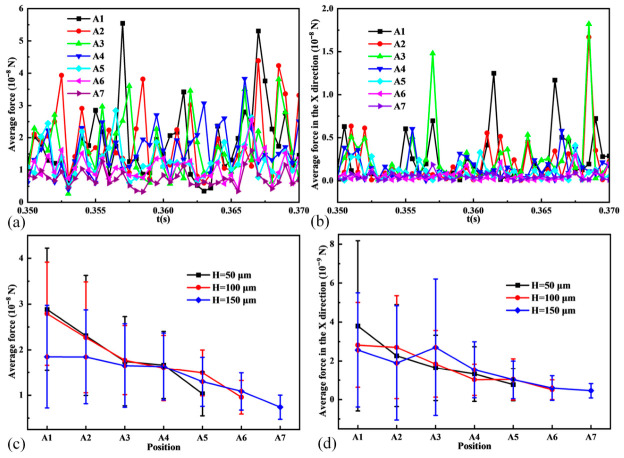
Analysis of forces within the area ahead of the blade for different layer thicknesses. (**a**) Temporal evolution of average total force in sub-areas A1–A7 (H = 150 µm). (**b**) Temporal evolution of average X-direction force magnitude in sub-areas A1–A7 (H = 150 µm). (**c**) Average total force in each sub-area for different H. (**d**) Average X-direction force magnitude in each sub-areas for different H.

To further analyze this shielding effect, the average forces on particles within the area ahead of the blade (defined in Figure 15c) were examined as a function of distance from the blade bottom. The sub-areas, each with dimensions of 200 µm, 500 µm, and 50 µm in the X, Y, and Z directions, respectively, are designated as A1 through A7. As the domain width in the Y-direction is exactly 500 µm, each sub-area extends across the full width of the computational domain. Thus, their distances from the bottom of the blade are 0 µm, 50 µm, 100 µm, 150 µm, 200 µm, 250 µm, and 300 µm, respectively. Figure 16a,b show the temporal evolution of average total force and X-direction force magnitude for H = 150 µm. Forces are highest and most variable close to the blade and decay rapidly with distance. Figure 16c compares the average total force in each sub-area for different H. The force in the critical near-blade region (A1, A2) is significantly lower for H = 150 µm compared to smaller thicknesses. This is primarily because a larger layer thickness provides a sufficient buffer zone between the blade and the fixed base, which weakens the strong interactions between particles and reduces occurrences such as particle clogging. Interestingly, Figure 16d shows that the average X-direction force within the pile itself (sub-areas A1–A7) is relatively insensitive to H, indicating that layer thickness primarily affects the interaction near the blade–bed interface rather than the internal dynamics of the spreading pile.

### 3.4. Powder Spreading Process Optimization

Synthesizing the aforementioned effects of spreading speed and layer thickness on powder bed quality, it can be summarized that these two parameters govern bed quality through different dominant mechanisms: spreading speed primarily regulates the deposition efficiency of the upper powder and the degree of disturbance to the underlying powder by influencing particle kinetic energy and the structure of the force chain network; layer thickness mainly controls the disturbance to the underlying powder by altering the efficiency of downward driving force transmission. In studies on the spreading of functionally graded materials, Wang et al. [28] also observed a similar phenomenon—higher spreading speeds exacerbate the diffusion of heavier particles into the region of lighter particles.

**Figure 17:**
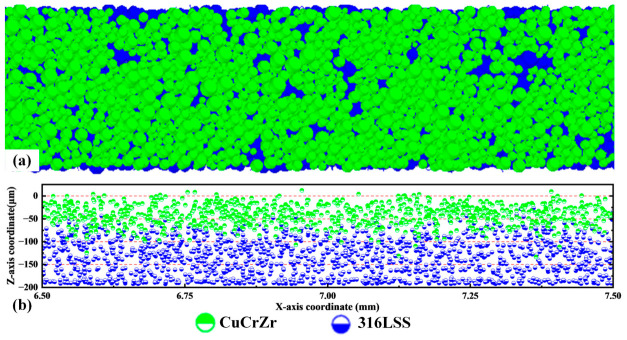
Powder bed particle distribution after spreading at v = 0.2 m/s and H = 150 µm. (**a**) Top view. (**b**) XZ plane projection.

In practical production, it is often desirable to use a higher spreading speed to achieve greater productivity; however, a high spreading speed is detrimental to the formation of a high-quality powder bed. The preceding analysis suggests that increasing layer thickness can mitigate the adverse effects of high speed. To explore this trade-off, a simulation was performed combining a relatively high speed (0.2 m/s) with a large layer thickness (150 µm). The resulting powder bed is shown in Figure 17. A substantial amount of CuCrZr is deposited, with a relatively uniform distribution. The CuCrZr particles predominantly occupy the upper region (Z ≈ −50 to 0 µm). Some CuCrZr particles are found deeper (−100 to −50 µm), intermixed with 316L SS, likely due to the higher kinetic energy at increased speed driving deeper penetration. Nevertheless, the region above −50 µm is almost exclusively CuCrZr, providing a relatively pure surface layer.

**Figure 18:**
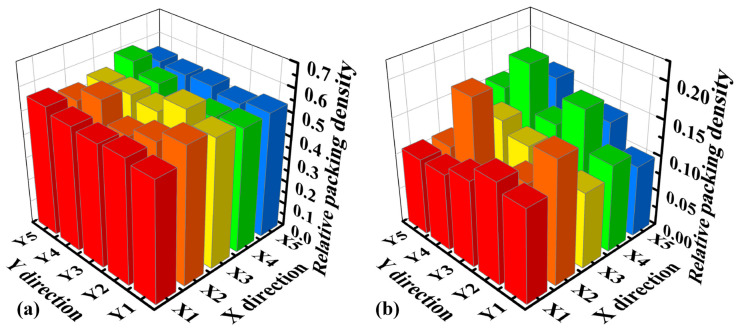
Quantitative analysis of powder bed quality for v = 0.2 m/s, H = 150 µm. (**a**) Spatial map of relative density for all particles. (**b**) Spatial map of relative density for CuCrZr-only.

The corresponding quantitative analysis is presented in Figure 18. The overall relative packing density reaches ~0.55, and the CuCrZr relative density is ~0.13. These values are comparable to those achieved with the slower speed (0.1 m/s) and moderate layer thickness (100 µm) combination (see Figure 14). Critically, the uniformity (indicated by the variation in the spatial maps in Figure 18a,b) is excellent. This demonstrates that the combination of higher speed and larger layer thickness can successfully reconcile the conflict between productivity and bed quality, enabling efficient spreading while maintaining a high-quality powder bed suitable for subsequent processing.

## 4. Conclusions

This study developed a Discrete Element Method (DEM) model to simulate the powder spreading process at a Z-direction heterogeneous material interface in multi-material laser powder bed fusion. The effects of spreading speed and theoretical layer thickness on the resulting powder bed quality were systematically investigated, leading to the following key conclusions:

(1) During the powder spreading process over a heterogeneous bed, the underlying powder particles, driven directly by the advancing upper powder and indirectly by the blade, exhibit a characteristic unsteady “forward-surging and rearward-suppressing” motion. This dynamic, coupled with significant spatiotemporal fluctuations in inter-particle force chains, leads to considerable randomness and inherent non-uniformity in the final particle distribution within the powder bed.

(2) Spreading speed critically influences bed quality. Increasing the speed from 0.05 m/s to 0.15 m/s significantly reduces the relative packing density of the upper material (CuCrZr) from ~0.11 to ~0.02 and also decreases the density of the underlying material (316L SS) from ~0.42 to ~0.38. Higher speeds intensify particle collisions and momentum transfer, enhancing the disturbance and entrainment of the underlying powder, which is detrimental to forming a high-quality, uniform bed.

(3) Increasing the theoretical layer thickness effectively mitigates the mechanical disturbance of the underlying layer. Raising the thickness from 50 μm to 150 μm increases the relative packing density of CuCrZr from ~0.04 to ~0.21, while the underlying 316L SS density remains stable (~0.38–0.41). A larger layer thickness provides ample deposition space for the incoming powder and, crucially, forms a thicker buffer zone between the blade and the underlying layer, absorbing mechanical energy and promoting stable, uniform deposition.

(4) The study demonstrates a viable strategy for process optimization. A combination of a higher spreading speed (0.2 m/s) and a larger layer thickness (150 µm) achieves an overall relative packing density of ~0.55 and a CuCrZr density of ~0.13, with spatial uniformity. This configuration successfully balances the competing demands of high spreading efficiency and superior powder bed quality, offering a practical reference for optimizing multi-material laser powder bed fusion processes.

## Figures and Tables

**Table 1:** Material physical properties used in DEM simulations.

Parameters	Values	
	316L SS	CuCrZr
Poisson’s ratio, σ	0.3	0.34
Density, ρ $/(\mathrm{kg}\cdot m^{3}$)	7800	8900
Young’s modulus, E/(GPa)	210	140
Restitution coefficient, e	0.9	0.88
Sliding friction coefficient, $\mu_{s}$	0.6	0.63
Rolling friction coefficient, $\mu_{r}$	0.085	0.09
Surface energy, γ $/(\mathrm{mJ}\cdot m^{-2}$)	0.097	0.1

## Data Availability

The raw data supporting the conclusions of this article will be made available by the authors on request.

## References

[1] Nazir A., Gokcekaya O., Md Masum Billah K., Ertugrul O., Jiang J., Sun J., Hussain S. (2023). Multi-material additive manufacturing: A systematic review of design, properties, applications, challenges, and 3D printing of materials and cellular metamaterials. Mater. Des..

[2] Liu Y., Liu G.T., Meng F.W., Tian D.B. (2024). An Overview of Multi-material Additive Manufacturing Processes. Aerosp. China.

[3] Clare A.T., Woizeschke P., Rankouhi B., Pfefferkorn F.E., Bartels D., Schmidt M., Wits W.W. (2025). Metal multi-material additive manufacturing: Overcoming barriers to implementation. CIRP Ann..

[4] Blakey-Milner B., Gradl P., Snedden G., Brooks M., Pitot J., Lopez E., Leary M., Berto F., du Plessis A. (2021). Metal additive manufacturing in aerospace: A review. Mater. Des..

[5] Arefin N., Moni H.-E.J., Espinosa D., Cong W., Zeng M. (2025). Multi-material additive manufacturing of energy storage and conversion devices: Recent progress and future prospects. Appl. Phys. Rev..

[6] Javidrad H., Koc B. (2025). Additively manufacturing of functionally graded multi-material parts using directed energy deposition. CIRP J. Manuf. Sci. Technol..

[7] Segovia-Guerrero L., Baladés N., Attard B., De Nicolás M., Scotti A., Zammit A., Sales D.L. (2024). Multi-material stainless steel fabrication using plasma wire arc additive manufacturing. J. Mater. Res. Technol..

[8] Liu L., Wang D., Wang T., Han C., Li Y., Tan H., Zhou W., Yan X., Lei L., Yang Y. (2025). Laser additive manufacturing of multimaterials with hierarchical interlocking interface via a flexible scraper-based method. Int. J. Mach. Tools Manuf..

[9] Chueh Y.-H., Hsieh B.-Y., Shih A.J. (2024). Interfacial characteristics in multi-material laser powder bed fusion of CuZr/316L stainless steel. CIRP Ann..

[10] Sing S.L., Huang S., Goh G.D., Goh G.L., Tey C.F., Tan J.H.K., Yeong W.Y. (2021). Emerging metallic systems for additive manufacturing: In-situ alloying and multi-metal processing in laser powder bed fusion. Prog. Mater. Sci..

[11] ZainElabdeen I.H., Ismail L., Mohamed O.F., Khan K.A., Schiffer A. (2024). Recent advancements in hybrid additive manufacturing of similar and dissimilar metals via laser powder bed fusion. Mater. Sci. Eng. A.

[12] Wang D., Liu L., Tang J., Liu Y., Wei C., Weng Z., Shao J., Tan H., Zhou W., Neirinck B. (2025). Recent advances on additive manufacturing of heterogeneous/gradient metallic materials via laser powder bed fusion. Int. J. Extrem. Manuf..

[13] Wang D., Liu L., Deng G., Deng C., Bai Y., Yang Y., Wu W., Chen J., Liu Y., Wang Y. (2022). Recent progress on additive manufacturing of multi-material structures with laser powder bed fusion. Virtual Phys. Prototyp..

[14] Tan C., Zhang X., Dong D., Attard B., Wang D., Kuang M., Ma W., Zhou K. (2020). In-situ synthesised interlayer enhances bonding strength in additively manufactured multi-material hybrid tooling. Int. J. Mach. Tools Manuf..

[15] Avrampos P., Vosniakos G.-C. (2022). A review of powder deposition in additive manufacturing by powder bed fusion. J. Manuf. Process..

[16] Talebi F.A., Haydari Z., Salehi H., Mehrabi M., Gardy J., Bradley M., Bayly A.E., Hassanpour A. (2024). Spreadability of powders for additive manufacturing: A critical review of metrics and characterisation methods. Particuology.

[17] Jump N., Sun P., Fang Z.Z. (2025). DEM modeling of particle size distribution and packing density and its application in Sinter-Based additive manufacturing. Prog. Addit. Manuf..

[18] Parteli E.J.R., Pöschel T. (2016). Particle-based simulation of powder application in additive manufacturing. Powder Technol..

[19] Chen H., Wei Q., Wen S., Li Z., Shi Y. (2017). Flow behavior of powder particles in layering process of selective laser melting: Numerical modeling and experimental verification based on discrete element method. Int. J. Mach. Tools Manuf..

[20] Chen H., Wei Q., Zhang Y., Chen F., Shi Y., Yan W. (2019). Powder-spreading mechanisms in powder-bed-based additive manufacturing: Experiments and computational modeling. Acta Mater..

[21] Chen H., Chen Y., Liu Y., Wei Q., Shi Y., Yan W. (2020). Packing quality of powder layer during counter-rolling-type powder spreading process in additive manufacturing. Int. J. Mach. Tools Manuf..

[22] Nan W., Ghadiri M. (2019). Numerical simulation of powder flow during spreading in additive manufacturing. Powder Technol..

[23] Nan W., Pasha M., Ghadiri M. (2020). Numerical simulation of particle flow and segregation during roller spreading process in additive manufacturing. Powder Technol..

[24] Chen H., Cheng T., Wei Q., Yan W. (2021). Dynamics of short fiber/polymer composite particles in paving process of additive manufacturing. Addit. Manuf..

[25] Yao D., Wang J., Cai Y., Zhao T., An X., Zhang H., Fu H., Yang X., Zou Q., Wang L. (2022). Composition regulation of composite materials in laser powder bed fusion additive manufacturing. Powder Technol..

[26] Zhang J., Huang G., Xu Y., Wang J., Han G., Tan Y. (2024). Dynamic simulation of powder spreading processes toward the fabrication of metal-matrix diamond composites in selective laser melting. Int. J. Refract. Met. Hard Mater..

[27] Tan P., Zhou M., Tang C., Zhou K. (2024). A powder-scale multiphysics framework for powder bed fusion of fiber-reinforced polymer composites. Adv. Powder Mater..

[28] Wang L., Li E., Zhou Z., Zhang B., Yu A. (2023). Simulation of powder spreading of functionally graded materials in powder bed fusion additive manufacturing. Acta Mech. Sin..

[29] Wischeropp T.M., Emmelmann C., Brandt M., Pateras A. (2019). Measurement of actual powder layer height and packing density in a single layer in selective laser melting. Addit. Manuf..

[30] Lu P., Chen-lin Z., Tong L., Jiang-lin L., Heng-hua Z., Mei Z. (2026). Exploring the actual stacking height of metal powder bed in laser powder bed fusion additive manufacturing. Sci. Rep..

[31] Wu Q., Zou Y., Kanishka S.A., Chiu L.N.S., Wu Y., Qiao C., Shen H., An X., Huang A. (2026). Challenges of powder spreading in laser powder bed fusion additive manufacturing of lattice structures: The phenomena, mechanisms, and solutions. J. Manuf. Process..

[32] Li Z., Mizutani M. (2023). Influence of layer thickness and substrate bed on the void fraction of powder layers for laser powder bed fusion. Powder Technol..

[33] Stephan M., Roux G., Burr A., Ablitzer C., Garandet J.-P. (2023). Identification of the influential DEM contact law parameters on powder bed quality and flow in additive manufacturing configurations. Powder Technol..

[34] Nan W., Pasha M., Ghadiri M. (2020). Effect of gas-particle interaction on roller spreading process in additive manufacturing. Powder Technol..

[35] Thornton C. (2015). Granular Dynamics, Contact Mechanics and Particle System Simulations.

[36] Parteli E.J.R., Schmidt J., Blümel C., Wirth K.-E., Peukert W., Pöschel T. (2014). Attractive particle interaction forces and packing density of fine glass powders. Sci. Rep..

[37] Yao D., Liu X., Wang J., Fan W., Li M., Fu H., Zhang H., Yang X., Zou Q., An X. (2021). Numerical insights on the spreading of practical 316 L stainless steel powder in SLM additive manufacturing. Powder Technol..

[38] O’Sullivan C., Bray J.D. (2004). Selecting a suitable time step for discrete element simulations that use the central difference time integration scheme. Eng. Comput..

[39] Nan W., Pasha M., Bonakdar T., Lopez A., Zafar U., Nadimi S., Ghadiri M. (2018). Jamming during particle spreading in additive manufacturing. Powder Technol..

[40] Du K., Li S., Jie S., Gao X., Yu Y. (2019). Effect of 316L stainless steel powder size distribution on selective laser melting process. J. Phys. Conf. Ser..

[41] Geer S., Bernhardt-Barry M.L., Garboczi E.J., Whiting J., Donmez A. (2018). A more efficient method for calibrating discrete element method parameters for simulations of metallic powder used in additive manufacturing. Granul. Matter.

[42] He Z., Nan W., Ma R., Ge L., He Y. (2025). Effects of particle shape on the jamming and rheological behavior of granular spreading flow. Phys. Fluids.

[43] Ren L., Ding L. (2025). Effects of cohesion on heterogeneous powder beds in additive manufacturing. Powder Metall. Ind..

[44] Das A., Derimow N., Tarr J., Hrabe N., Weaver J. (2025). Understanding the effects of metal powder feedstock heterogeneity on the laser powder bed fusion process. Materialia.

[45] Yao L., Xiao Z., Hoo Z., Tang C., Qiao J., Zhang Y. (2023). Mechanism analysis of grain growth dominated by alloy composition gradients during powder bed fusion. Mater. Res. Lett..

[46] Samaei A., Sang Z., Glerum J.A., Mogonye J.-E., Wagner G.J. (2023). Multiphysics modeling of mixing and material transport in additive manufacturing with multicomponent powder beds. Addit. Manuf..

[47] Nan W., Ge L., Xuan W., Gu Y. (2024). Transient jamming of granular flow by blade spreading. Powder Technol..

[48] Avrampos P., Vosniakos G.-C. (2024). A Study on Powder Spreading Quality in Powder Bed Fusion Processes Using Discrete Element Method Simulation. J. Manuf. Mater. Process..

